# An Account of Diseases in an Eastern District of London, from the 20th of July, to the 20th of August, 1811

**Published:** 1811-09

**Authors:** 


*An Account of Diseases in an Eastern District of London, from the 20th of July, to the 20th of August,* 1811.

ACUTE DISEASES.

| | |
|---|---|
| Typhus mitior | 5 |
| Scarlatina | 3 |
| Scarlatina Anginosa | 5 |
| Rheumatismus acutus | 3 |

CHRONIC DISEASES.

| | |
|---|---|
| Tussis | 7 |
| Dyspnœa | 4 |
| Tussis cum Dyspnœa | 5 |
| Hydrothorax | 3 |
| Phthisis Pulmonalis | 4 |
| Peripneumonia notha | 3 |
| Pleurodyne | 2 |
| Ascites | 1 |
| Diarrhæa | 5 |
| Menorrhagia | 4 |
| Dysuria | 3 |
| Leucorrhœa | 6 |
| Hæmorrhagia Intestinalis | 2 |
| Rheumatismus Chronicus | 6 |

PUERPERAL DISEASES.

| | |
|---|---|
| Menorrhagia Lochialis | 7 |
| Ephemera | 3 |
| Mastodynia | 4 |
| Dolor post partum | 6 |

INFANTILE DISEASES.

| | |
|---|---|
| Ophthalmia Purulenta | 5 |
| Dentitio | 6 |
| Hydrocephalus Internus | 1 |

For several weeks past there has been a very frequent recurrence of febrile diseases, which, as it was remarked in a former report, had very seldom appeared during a few preceding years. Scarlatina has been a prevailing disease amongst children. It has appeared more generally in the surrounding villages than in the metropolis. Where it has found admission into families consisting principally of young children, it has generally spread through the whole of them, and, in many instances, has proved fatal in its consequences. When this disease appears with milder symptoms, and without any affection of the throat. it frequently gives way to a moderately cooling treatment; but when a high degree of fever prevails with considerable swelling of the tonsils, attended with difficult and painful deglutition, a more strictly antiphlogis[illegible] treatment is necessary, especially at the commencement of the d[illegible]ease. To what extent this part of the medical practice should be carried, can be determined only by the urgency of the symptoms and the various circumstances under which they occur. It is by a proper discrimination in some of these critical cases, and by an accurate decision, that the sagacity of the medical attendant is discovered.

In a disease which sometimes makes a rapid progress through its stages, and is ultimately attended with symptoms of putrescency, it has perhaps been too common a practice, in order to obviate these, to commence the cordial plan of cure too early, and thus eventually to accelerate the progress of symptoms which it was designed to retard.

